# Assessment of WOMAC index for knee osteoarthritis and its association with serum vitamin - D and serum cytokine profile

**DOI:** 10.6026/973206300211791

**Published:** 2025-07-31

**Authors:** Deepak Kumar, Mithilesh Kannaujiya, Shailendra Singh, Sanjeev Kumar, Shah Waliullah, Sujeet Kumar Chaudhary, Ashish Kumar

**Affiliations:** 1 Department of Orthopaedic Surgery, King George's Medical University, Lucknow, India

**Keywords:** Vitamin D, osteoarthritis, IL-6, TNF-α, WOMAC, inflammation, cytokines

## Abstract

Vitamin D has immuno-modulatory and potential cartilage-regenerative effects. Therefore, it is of interest to evaluate the association between serum vitamin D levels, pro-inflammatory cytokines (IL-6, TNF-α), and functional status in patients with knee osteoarthritis (OA). In this cross-sectional analysis of 84 patients with bilateral knee OA, serum vitamin D, IL-6, TNF-α levels and WOMAC scores were recorded. Patients with vitamin D deficiency exhibited significantly higher IL-6 and TNF-α levels and worse WOMAC scores. Data shows that vitamin D deficiency is associated with increased inflammation and poorer functional outcomes in knee OA.

## Background:

Vitamin - D deficiency is prevalent and linked to increased knee pain, osteoarthritis prevalence, and progression of knee osteoarthritis [1, 2]. Vitamin - D is known to increase bone mass or prevent bone loss. Thus, older adults are recommended to consume adequate vitamin - D to prevent osteoporosis and fracture [3]. For instance, the use of high-dose vitamin - D supplements (≥1000 International Units (IU)/d) is increasing in the United States (US) with over 30% of adults aged ≥60 years consuming ≥1000 IU/d through supplementation [4]. Globally 9.6% of men and 18.0% of women aged over 60 years have symptomatic osteoarthritis. Eighty percent of those with osteoarthritis have limitations in movement and 25% cannot perform their major daily activities of life [5]. Vitamin - D's immune-regulating properties help combat inflammation by stimulating the production of anti-inflammatory cytokines and reducing proinflammatory ones. It may also promote cartilage regeneration, according to emerging evidence [6]. Osteoarthritis is caused by an imbalance of proinflammatory cytokines, leading to a vicious cycle of damage to cartilage and intra-articular structures, activated by matrix metalloproteinases (MMPs) and ADAMTS, a disintegrin-like and metalloproteinase with thrombospondin motif [7]. The most important inflammatory mediators in the pathogenesis of OA are IL-1β, TNF-α and IL-6. They are activators of different signalling pathways that activate other cytokines and pathologic processes, this unstoppable process are chemokines that, stimulated by cytokines, attract inflammatory cells to the joint that further promote the secretion of inflammatory factors and disease progression [8]. Synovial inflammation is increasingly linked to disease progression, with inflammatory molecules like IL-6 and TNF-an implicated. Elevated IL-6 levels in synovial fluid in patients with focal cartilage defects increase the likelihood of radiographic knee OA [9, 10]. In an experimental canine model, high concentrations of IL-6 and TNF-a were associated with early OA [11]. Therefore, the use of nutraceuticals might be particularly interesting for the action on pain, chronic in OA and represents the main cause of disability for this condition. For evaluation of OA pain intensity, the most common criteria include the visual analog scale (VAS) or numerical rating scale (NRS), the pain subscale of the Western Ontario and McMaster Universities Arthritis Index (WOMAC), and the Knee injury and Osteoarthritis Outcome Score (KOOS) [12]. In particular, the WOMAC index is widely used in the evaluation of hip and knee osteoarthritis based on a self-administered questionnaire consisting of items regarding pain, stiffness, and physical function. In this regard, a recent meta-analysis of 42 random clinical trials (RCTs) utilizing nutraceuticals (such as chondroitin sulphate, glucosamine sulphate, collagen, and hyaluronic acid) found improvements across all OA measurement parameters expressed through the total WOMAC index, WOMAC pain and WOMAC stiffness subscales, and VAS [13]. Therefore, it is of interest to explore the correlation between serum vitamin - D levels and cytokine profiles (IL-6 and TNF ALPHA) in patients with knee osteoarthritis, using clinical assessment with the WOMAC score.

## Methods:

In this Prospective cross-sectional Analytical study the material for the study was collected from Department of Orthopaedics, in collaboration with Department of Biochemistry, King Georges Medical University, Lucknow, in a period of one year. Total 84 patients were included with complain of bilateral osteoarthritis knee with age more than 50 years. Levels of interleukins -6 and TNF alpha were measured by ELISA. Additionally, WOMAC scoring was conducted to evaluate the patients' condition. The study included patients over 50 years old with radiologic diagnosis of grade I to III primary knee OA and bilateral involvement, while exclusion criteria included patients with Kellgren and Lawrence grade IV, concomitant disorders affecting serum cytokines levels, chronic inflammatory disorders, infection, diabetes mellitus, cardiovascular disorders, renal disorders, and neurodegenerative disorders.

## Statistical analysis:

The study used Statistical Package for Social Sciences (SPSS) version 23.0 to analyze data, with continuous variables represented as mean ± standard deviation and categorical variables as percentages. The chi-square test and student-t test were used for comparison of nominal data, with p < 0.05 as the cut off value.

## Observation and Results:

The study included 84 patients, with the majority aged 51-60 years. The mean age was 61.33±7.81 years. Out of the patients, 48 (57.1%) were male, while 36 (42.9%) were female. The majority of patients were in Kellgren and Lawrence grade II, with 48.8% in Grade II. The remaining patients were in Grade I, 17.9% in Grade I, and 33.3% in Grade III. Only one patient was 81 years or older (Table 1 - see PDF). The mean serum vitamin - D level among the patients was 28.72±11.87 ng/ml. Additionally, the mean levels of IL-6 and TNF ALPHA cytokines were reported as 172.14±215.37 pg/ml and 132.52±167.80 pg/ml, respectively. Moreover, the mean WOMAC-Index was reported as 71.60±9.38% (Table 2 - see PDF). Out of 84 patients studied, 27 (32.1%) patients had normal vitamin - D levels and a mean vitamin - D level of 43.42±9.01 ng/ml. Additionally, 36 s (42.9%) patients had scant vitamin - D levels, ranging from 20 to 29 ng/ml, and a mean level of 24.24±2.92 ng/ml. Furthermore, 21 (25.0%) patients were categorized as having a deficiency in vitamin - D, defined as 20 ng/ml or lower, with a mean level of 17.51±2.18 ng/ml (Table 3 - see PDF). The study found a significant association between serum vitamin - D levels and IL-6 concentrations in patients with knee osteoarthritis. Patients with normal vitamin - D levels had a mean IL-6 concentration of 94.65±34.77 pg/ml, while those with scant vitamin - D levels (20-29 ng/ml) had a higher mean IL-6 concentration of 140.65±30.85 pg/ml. Patients with vitamin - D deficiency (20 ng/ml and lower) had the highest mean IL-6 concentration of 325.74±393.19 pg/ml. The mean TNF ALPHA concentration was also higher in patients with normal vitamin - D levels (30 ng/ml and above) and those with scant vitamin - D levels (20-29 ng/ml). The mean WOMAC Index score was also higher in patients with vitamin - D deficiency (20 ng/ml and lower); with the highest mean score of 81.39±6.84. The study highlights the importance of maintaining adequate vitamin - D levels in patients with knee osteoarthritis (Table 4 - see PDF). It was observed that as KL grades increase the level of vitamin - D decreases and there was a statistically significant strong association between serum vitamin - D levels and IL-6 concentrations in patients with knee osteoarthritis (p<0.05) (Table 5, 6 - see PDF).

## Discussion:

Vitamin D deficiency and knee osteoarthritis (OA) are major global health concerns, particularly among the elderly. Subchondral bone alterations are central to cartilage degeneration, and low levels of serum 25-OH vitamin D are linked to increased osteoblastic activity and bone turnover. The progression of OA is influenced by enhanced bone resorption due to elevated parathyroid hormone levels, increased bone turnover, and the direct action of vitamin D metabolites on articular chondrocytes [2]. Early structural changes in the joint, including cartilage defects, subchondral bone expansion, and bone marrow lesions, may occur before the clinical onset of OA. Measuring serum vitamin D levels in elderly individuals with OA and providing supplementation to restore adequate levels has been recommended to potentially delay disease progression [2, 3]. Pro-inflammatory cytokines, particularly IL-6 and TNF-α, play a significant role in OA pathogenesis. Stannus *et al.* reported that serum levels of IL-6 and TNF-α were significantly associated with joint space narrowing (JSN) in the medial tibiofemoral compartment and predicted loss of tibial cartilage volume over time [14]. These findings support the inflammatory basis of OA and the potential for anti-inflammatory interventions. In the present study, the majority of patients were aged 51-60 years (mean age 61.33±7.81), with male predominance (57.1%), consistent with the demographic pattern reported by Anari *et al.* and Stannus *et al.* [14, 15]. However, Namutebi *et al.* and Kadijani *et al.* found a female predominance in their OA populations [16, 17]. Our findings showed that 48.8% of patients were classified as Kellgren and Lawrence grade II, similar to the distribution reported by Anari *et al.* [15] and Barkera *et al.* [18]. The mean serum vitamin D level in our cohort was 28.72±11.87 ng/ml, in line with studies by Kadijani *et al.* (33.5±18.1 ng/ml) and Namutebi *et al.* (35.5% sufficient, 50.5% insufficient, 14.0% deficient) [16, 17]. Serum IL-6 and TNF-α levels in our patients were markedly elevated (172.14±215.37 pg/ml and 132.52±167.80 pg/ml, respectively), significantly higher than those reported by Kadijani *et al.* (IL-6: 6.5±2.5 pg/ml; TNF-α: 40.2±14.7 pg/ml) [17], suggesting a more inflammatory phenotype or potentially more advanced disease stage in our study group. Our study revealed a strong inverse association between vitamin D levels and WOMAC scores. Patients with sufficient vitamin D levels had the lowest WOMAC scores (63.12±5.67), while those with deficiency (<20 ng/ml) had significantly higher scores (81.39±6.84), indicating worse pain and function. Kadijani *et al.* similarly observed a negative correlation between IL-6 and vitamin D levels (r = -0.220, p = 0.018), supporting this relationship. These findings underline the importance of vitamin D in modulating inflammation and functional outcomes in OA. Elevated IL-6 has been associated with both knee and hip OA and correlates with pain intensity, unlike IL-10 which showed no such correlation [17]. Despite its strengths, this study has limitations. Only serum cytokine markers (IL-6 and TNF-α) were analyzed, without exploring the broader panel of inflammatory markers or synovial fluid levels. Additionally, it lacked a longitudinal follow-up to evaluate the effect of vitamin D supplementation over time.

## Conclusion:

Vitamin D deficiency is associated with elevated inflammatory cytokines (IL-6 and TNF-α) and worse clinical outcomes in knee osteoarthritis. Patients with sufficient vitamin D levels showed lower WOMAC scores, indicating better function. Data suggest a potential role for vitamin D supplementation in reducing inflammation and disease severity in knee OA.
